# An Account of the Practice of One of the Physicians of the Westminster General Dispensary, and of the Western Dispensary, from the 20th of November, to the 20th of December, 1807

**Published:** 1808-01

**Authors:** Samuel Fothergill

**Affiliations:** Southampton-Street, Strand


### *An Account of the Practice of one of the Physicians of the Westminster General Dispensary, and of the Western Dispensary, from the 20th of November, to the 20th of December, 1807.*

| | | | |
|---|---|---|---|
| ACUTE DISEASES. | | Pneumonia Notha - | 2 |
| Catarrh - - | 11 | Asthma - - - - | 4 |
| Measles - - | 9 | Chronic Rheumatism | 5 |
| Hooping-Cough - | 4 | Hydrocephalus Internus | 2 |
| Small-pox - - | 6 | Ophthalmia - - | 1 |
| Scarlatina Anginosa - | 4 | Inflammation of the Liver | 2 |
| Inflammatory Sore-Throat | 2 | Dropsy - - - | 5 |
| Typhus - - - | 1 | Asthenia - - | 9 |
| Acute Rheumatism - | 6 | Dysentery - - | 2 |
| Acute Diseases of Infants | 12 | Vomiting of Blood - | 1 |
| CHRONIC DISEASES. | | Bilious Vomiting - | 2 |
| Cough and Dyspnœa | 20 | Diarrhœa - - | 3 |
| Pulmonary Consumption | 4 | Dyspepsia - - | 4 |
| Hæmoptoe - | 2 | Jaundice - - | 3 |

Gastrodynia

| | | | |
|---|---|---|---|
| Gastrodynia | 2 | Psora | 1 |
| Paralysis | 3 | Menorrhagia | 2 |
| Dysury | 2 | Amenorrhœa | 4 |
| Porrigo | 2 | Leucorrhœa | 3 |

The winter has began early, and with more than usual severity. The proportion of Acute Diseases, as will appear from the present list, is already augmented. For some time past, measles have been very frequent; and several children have died from that disease. In some of these, effusion had taken place within the chest; and in some, the inner membrane of the trachea was inflamed with a layer of coagulable lymph and mucous upon its surface. Of the nine cases enumerated in this report, one terminated fatally; it was accompanied from the time of the eruption disappearing with a most distressing and painful difficulty of breathing.

In one family three young children, who had struggled for four months with hooping-cough, and were reduced to a deplorable state, became affected with measles. One of these, about two years old, suffered much; the cough was hard, the breathing very quick and painful; the face flushed, fever high, and a pulse at one period not to be numbered from its frequency. The abdominal muscles assisting those more immediately subservient to respiration, contracted forcibly, and the rectum was protruded, which, becoming inflamed, occasioned considerable uneasiness. Notwithstanding these formidable symptoms, the treatment was successful; but the infant being in a state of great debility, confined in a vitiated atmosphere, and not experiencing those attentions which, perhaps, affluence only could have afforded, was shortly after attacked with Hydrocephalus Internus, which in five days proved fatal. In this, and similar cases, where respiration is frequent and obstructed, the pulse very quick, the cheek suffused with a circular blush, and the patient at the same time affected with a distressing cough and little expectoration; in short, where effusion in the chest or pleuritic inflammation might speedily be apprehended, I have observed great relief follow the exhibition of antimony and fox-glove; which reduce the increased action, either by their immediate influence upon the vascular system, or by occasioning nausea and even vomiting; at the same time, purgatives are not to be omitted. In such cases, many children are lost from their inability to expectorate the mucous which is secreted within the trachea and lungs; this produces so much much irritation, that a general spasmodic and convulsive action of the muscles takes place; and unless the medicines usually employed in these extreme cases, be administered with a bold and decided hand, in much larger doses than timidity or inexperience would deem compatible with safety, they inevitably die.

SAMUEL FOTHERGILL.

*Southampton-Street, Strand.*

